# Inhibition of bacterial α-, β- and γ-class carbonic anhydrases with selenazoles incorporating benzenesulfonamide moieties

**DOI:** 10.1080/14756366.2018.1547287

**Published:** 2018-12-27

**Authors:** Andrea Angeli, Mariana Pinteala, Stelian S. Maier, Sonia Del Prete, Clemente Capasso, Bogdan C. Simionescu, Claudiu T. Supuran

**Affiliations:** a Dipartimento Neurofarba, Sezione di Scienze Farmaceutiche e Nutraceutiche, Università degli Studi di Firenze, Florence, Italy;; b Centre of Advanced Research in Bionanoconjugates and Biopolymers Department, “Petru Poni” Institute of Macromolecular Chemistry, Iasi, Romania;; c Polymers Research Center, Polymeric Release Systems Research Group, “Gheorghe Asachi” Technical University of Iasi, Iasi, Romania;; d Istituto di Bioscienze e Biorisorse, CNR, Napoli, Italy

**Keywords:** Carbonic anhydrase, bacterial enzymes, *Helicobacter pylori*, *Vibrio cholerae*, *Burkholderia pseudomallei*

## Abstract

A series of benzenesulfonamides incorporating selenazoles with diverse substitution patterns were investigated as inhibitors of six bacterial carbonic anhydrases (CAs, EC 4.2.1.1) from bacterial pathogens, such as *Helicobacter pylori* (hpCAα was the investigated enzyme), *Vibrio cholerae* (all the three CAs from this pathogen were considered, VchCAα, VchCAβ and VchCAγ) and *Burkholderia pseudomallei* (with its two CAs, BpsCAβ and BpsCAγ). All these sulfonamides were effective CA inhibitors, with potencies in the low micromolar or submicromolar range, making them attractive as lead compounds for designing antibacterials with a novel mechanism of action, which could counteract the extensive resistance problem observed with many clinically used antibiotics.

## 1. Introduction

**Figure 1:**
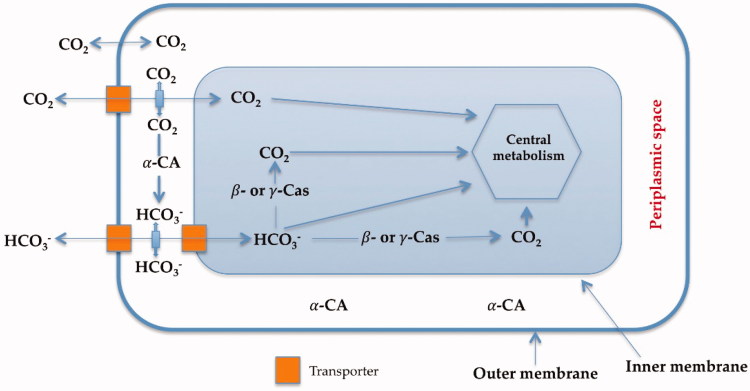
Proposed role of CAs in Gram-negative bacteria. The α-CAs convert the diffused CO_2_ inside the periplasmic space into bicarbonate, whereas the cytosolic β-and γ-CAs are responsible for the supplementation of CO_2_ and bicarbonate for the cellular metabolism. Furthermore, all these enzymes play an important role in the cellular pH homeostasis^1^.

Carbonic anhydrases (CAs, EC 4.2.1.1) are a superfamily of ubiquitous metalloenzymes which catalyze the simple but physiologically crucial interconversion of carbon dioxide and water, with the formation of bicarbonate and protons: CO_2_ + H_2_O ⇌ HCO_3_^−^ + H^+^^1–11^. The hydration/dehydration of CO_2_ is a key physiological reaction for the life cycle of most organisms, including bacteria, since it is connected with numerous metabolic pathways, such as the biosynthetic processes requiring CO_2_ or HCO_3_^−^ (photosynthesis and carboxylation reactions) and biochemical pathways including pH homeostasis, secretion of electrolytes, transport of CO_2_ and bicarbonate, etc.^12^^,^^13^. Figure 1 shows the transport of carbon dioxide and bicarbonate assisted by bacterial CAs denoting their pivotal role in the bacterial metabolism. In fact, bacteria encode such enzymes belonging to three different genetic families, the α-, β- and γ-CAs^11–13^.

Inhibition of one or more of the CAs present in bacteria was shown to interfere with the growth and/or pathogenicity of many such organisms^1–5^^,^^13^. The reason for that is explained as follows: these enzymes participate in tightly controlled metabolic processes, or in the pH homeostasis, which are highly relevant in all organisms, including bacteria^1–13^. It has been demonstrated that the two CAs (α and β) encoded by the genome of the pathogen *Helicobacter pylori,* a Gram-negative bacterium colonizing the human stomach, are essential for the acid acclimatization of the pathogen within the human stomach and thus, for the bacterial survival in the host^14^. On the other hand, *Vibrio cholerae* (a Gram-negative bacterium provoking cholera) uses its CAs (α, β and γ) for producing sodium bicarbonate, which induces cholera toxin expression^15^, and for colonizing the host^16^. Once more, the causative agent of brucellosis, *Brucella suis*, a non-motile Gram-negative coccobacillus, and the *Mycobacterium tuberculosis*, an obligate pathogenic bacterium responsible for tuberculosis, were needed of functional CAs for growing^17–19^. Indeed, a large number of interesting studies were dedicated in the last decade for finding effective *in vitro* CA inhibitors (CAIs) targeting these pathogenic enzymes^8–14^^,^^17–19^, and possibly translating such results to *in vivo* and eventually clinical activity^1^. However, CAIs are not yet seriously considered as potential anti-infectives to date, mainly due to the fact that no relevant drug discovery program has been yet started, although these compounds may show indeed a great promise for fighting drug resistant microbes, such as bacteria, fungi and protozoa^20^.

## 2. Materials and methods

### 2.1. Chemistry

Selenazoles **1**, **4–5** and **8–11** were reported earlier by our group and were used as follows^21^.

### 2.2. CA enzyme inhibition assay

An Sx.18Mv-R Applied Photophysics (Oxford, UK) stopped-flow instrument has been used to assay the catalytic activity of various CA isozymes for CO_2_ hydration reaction^22^. Phenol red (at a concentration of 0.2 mM) was used as an indicator, working at the absorbance maximum of 557 nm, with 10 mM Hepes (pH 7.5, for α-CAs) or TRIS (pH 8.3, for β- and γ-CAs) as buffers, 0.1 M Na_2_SO_4_ (for maintaining constant ionic strength), following the CA-catalyzed CO_2_ hydration reaction for a period of 10 s at 25 °C. The CO_2_ concentrations ranged from 1.7 to 17 mM for the determination of the kinetic parameters and inhibition constants. For each inhibitor, at least six traces of the initial 5–10% of the reaction have been used for determining the initial velocity. The uncatalyzed rates were determined in the same manner and subtracted from the total observed rates. Stock solutions of inhibitors (10 mM) were prepared in distilled-deionized water and dilutions up to 1 nM were done thereafter with the assay buffer. Enzyme and inhibitor solutions were pre-incubated together for 15 min (standard assay at room temperature) prior to assay, in order to allow for the formation of the enzyme–inhibitor complex. The inhibition constants were obtained by non-linear least-squares methods, using GraphPad PRISM 3 and the Cheng–Prusoff equation, as reported earlier^23^. All CAs were recombinant proteins produced as reported earlier by our groups^9–14^^,^^24^.

## 3. Results and discussion

### 3.1. Chemistry

**Scheme 1:**
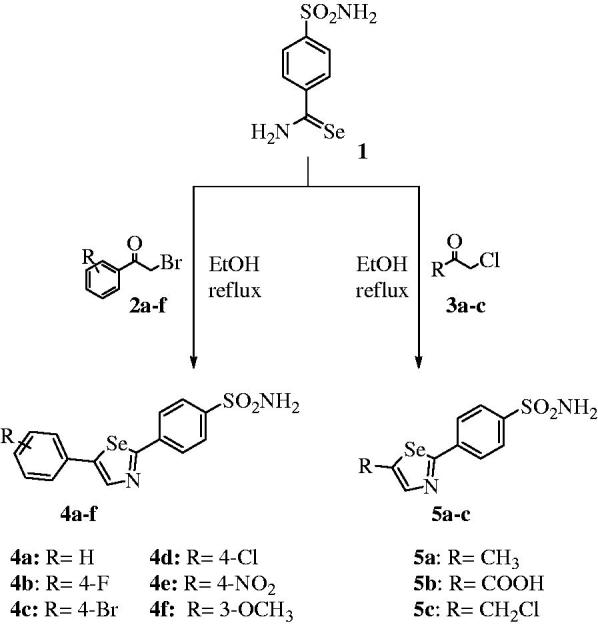
Synthesis of functionalized selenazoles **4a–f** and **5a–c**.

Selenazoles are an important class of heterocycles with significant biological effects and considerable pharmacological relevance^21^^,^^25^. Moreover, these five-membered selenium heterocycles are easily synthesized from primary selenoamide bearing benzenesulfonamide **1** as starting materials. This compound has been used for the preparation of various 2,5-disubstituted 1,3-selenazoles **4a-f** and **5a-c**, as reported earlier (Scheme 1)^21^.

**Scheme 2:**
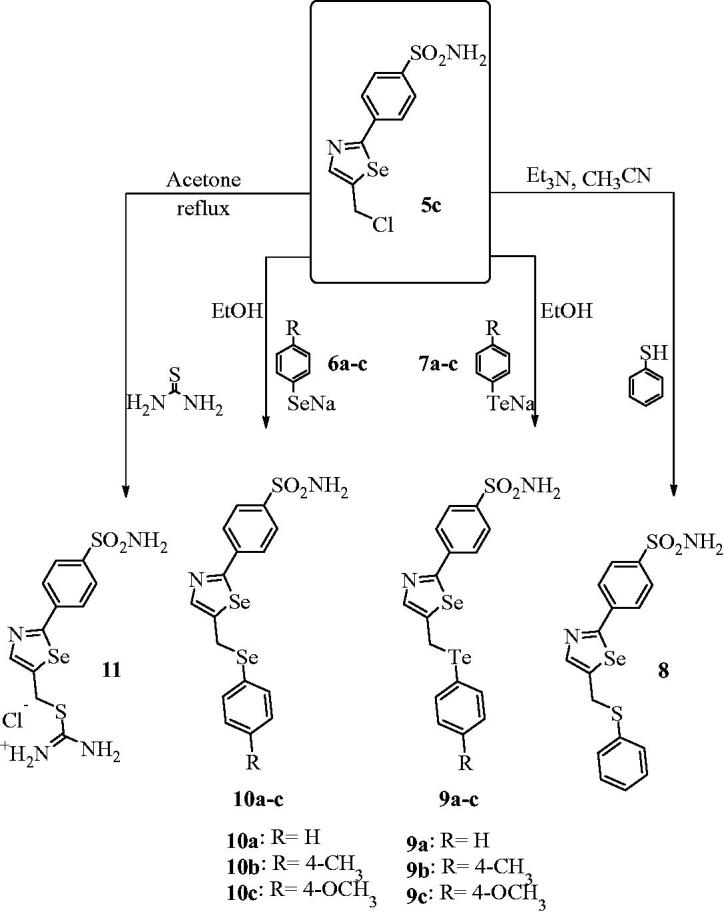
Synthesis of substituted 2,5-selenazoles **8**–**11**.

In order to extend our library of selenazole derivatives, we report the synthesis of a variety of double-functionalized and ionic 1,3-selenazoles, as shown in Scheme 2.

Our main interest was to investigate structure–activity relationship related to the inhibition of different CAs classes from three different pathogenic bacteria: *Helicobacter pylori* (hpCAα), *Vibrio cholerae* (VchCAα, VchCAβ and VchCAγ) and, *Burkholderia pseudomallei* (BpsCAβ and BpsCAγ), relevant pathogens in many diseases for which drug-resistant strains were evidenced^1^^,^^9–11^.

### 3.2. Carbonic anhydrase inhibition

Selenazole scaffolds showed promising results as antibacterial activity^26–28^. For this reason, we investigated whether such compounds may act as inhibitors of bacterial CAs, thus being possible candidates for anti-infective studies. We tested *in vitro* compounds **4–11** for their inhibitory activity against six bacterial enzyme: hpCAα, VchCAα, VchCAβ, VhCAγ, BpsCAβ and BpsCAγ, by means of the stopped-flow carbon dioxide hydration assay^22^. These activities were compared with those of the standard, clinically used CAI acetazolamide (**AAZ**) (Table 1).

**Table 1.** Inhibition data against bacterial enzyme hpCAα, VchCAα, VchCAβ, VchCAγ, BpsCAβ and BpsCAγ of derivates **4–5**, **8–11** and acetazolamide **AAZ** by a stopped-flow CO_2_ hydrase assay^22^.

	*K_I_* (µM)^a^					
Compound	hpCAα	VchCAα	VchCAβ	VchCAγ	BpsCAβ	BpsCAγ
**4a**	1.95	0.22	2.61	8.23	0.58	0.82
**4b**	2.11	0.85	6.54	4.11	0.80	0.91
**4c**	0.79	0.57	9.08	8.07	0.43	0.83
**4d**	2.03	0.71	8.60	5.72	0.65	0.67
**4e**	2.21	0.82	8.89	8.52	0.68	0.80
**4f**	1.73	0.92	8.40	5.83	0.62	4.39
**5a**	2.11	0.58	5.88	8.28	0.55	3.98
**5b**	1.64	0.27	8.95	9.29	0.36	7.00
**5c**	1.63	0.21	7.75	3.64	0.33	3.79
**8**	2.71	0.93	6.42	6.80	4.60	6.80
**9a**	2.72	0.94	7.70	8.90	0.92	8.90
**9b**	2.42	0.47	7.72	7.60	0.32	7.60
**9c**	2.84	0.77	9.08	6.23	5.50	6.23
**10a**	2.43	0.87	8.84	4.70	0.09	4.70
**10b**	2.43	0.46	8.59	8.74	4.69	8.74
**10c**	2.80	0.92	6.79	7.00	6.20	0.70
**11**	2.04	0.44	9.12	6.05	0.64	6.55
**AAZ**	0.02	0.007	0.45	0.47	0.74	0.15

^a^Mean from three different assays, by a stopped-flow technique (errors were in the range of ±5–10% of the reported values).

As seen from data of Table 1, all bacterial enzymes investigated here showed inhibition in the low micromolar or submicromolar range, with all selenazoles incorporating benzenesulfonamide mioietes included in the study.

(i) HpCAα was effectively inhibited, with K_I_s ranging between 0.79 and 2.84 µM, thus leaving little space to structure–activity relationship (SAR) discussion due to this flat profile. However, all these sulfonamides were at least two orders of magnitude less effective as HpCAα inhibitors compared to acetazolamide, which is a very potent inhibitor of this enzyme.

(ii) VchCAα was inhibited in the submicromolar range by selenazoles **4–11**, with *K_I_*s ranging between 0.21 and 0.94 µM, again without any relevant SAR, since the activity was very similar for the entire series of derivatives. However, the β- and γ-class enzymes from the same pathogen had a weaker sensitivity to this class of CAIs, since the *K_I_*s ranged between 2.61–9.12 µM for VchCAβ, and 3.64–9.29 µM for VchCAγ (Table 1). Again, **AAZ** was a better inhibitor for all these CAs compared to the studied derivatives.

(iii) Some of the selenazole sulfonamides investigated here were, on one hand, more effective BpsCAβ inhibitors compared to the standard drug **AAZ**, as they showed submicromolar inhibitory power. Among them were **10a** (K_I_ of 90 nM), **9b** (K_I_ of 320 nM), **5b** and **5c** (*K_I_*s of 0.33–0.36 µM), compared to the *K_I_* of 0.74 µM of acetazolamide. These data show that small structural differences lead to drastic effects on the CA inhibition. For example, **10b**, possessing an extra methyl group compared to **10a**, was 52 times a weaker BpsCAβ inhibitor compared to **10a**. BpsCAγ was, on the other hand, less sensitive to these inhibitors compared to the β-class enzyme from the same pathogen, although some compounds (**4a**–**4e**, **10c**) were submicromolar inhibitors, with *K_I_*s ranging between 0.70 and 0.91 µM.

## 4. Conclusions

A series of benzenesulfonamides incorporating selenazoles with diverse substitution patterns were investigated as inhibitors of six bacterial CAs from pathogens such as *Helicobacter pylori* (tested enzyme was hpCAα), *Vibrio cholerae* (all the three CAs from this pathogen were considered, VchCAα, VchCAβ and VchCAγ) and *Burkholderia pseudomallei* (with its two CAs, BpsCAβ and BpsCAγ). All these sulfonamides were effective inhibitors, with potencies in the low micromolar or submicromolar range, making this class of CA inhibitors attractive as lead compounds for designing antibacterials with a novel mechanism of action, which could counteract the extensive antibiotic resistance problem encountered with most clinically used such drugs.
